# An Application of Graphical Approach to Construct Multiple Testing Procedures in a Hypothetical Phase III Design

**DOI:** 10.3389/fpubh.2013.00075

**Published:** 2014-01-07

**Authors:** Bushi Wang, Naitee Ting

**Affiliations:** ^1^Biometrics, Boehringer-Ingelheim Pharmaceuticals, Inc., Ridgefield, CT, USA

**Keywords:** multiple testing procedures, non-inferiority, dose selection, multiple endpoints, superiority and non-inferiority

## Abstract

Many multiple testing procedures (MTP) have been developed in recent years. Among these new procedures, the graphical approach is flexible and easy to communicate with non-statisticians. A hypothetical Phase III clinical trial design is introduced in this manuscript to demonstrate how graphical approach can be applied in clinical product development. In this design, an active comparator is used. It is thought that this test drug under development could potentially be superior to this comparator. For comparison of efficacy, the primary endpoint is well established and widely accepted by regulatory agencies. However, an important secondary endpoint based on Phase II findings looks very promising. The target dose may have a good opportunity to deliver superiority to the comparator. Furthermore, a lower dose is included in case the target dose may demonstrate potential safety concerns. This Phase III study is designed as a non-inferiority trial with two doses, and two endpoints. This manuscript will illustrate how graphical approach is applied to this design in handling multiple testing issues.

## Introduction

There has been a rapid development in multiple testing procedures (MTP) in the past 10 years. Numerous of new procedures have been proposed to deal with not only multiple testing problems with single source of multiplicity, but also more complex multiple testing problems involving more than one source of multiplicity. For example, the presence of multiple endpoints, multiple dose regimens, non-inferiority and superiority tests, and multiple region or patient populations.

To meet the demand of more complex trial design and strong control of family-wise error rate, several papers have been published in recent years to construct powerful yet flexible MTP. For a summary of recent question and development in multiplicity issues, one can refer to Hung and Wang (1) and Wang and Cui (2).

In many cases, the need to design a complex trial is inevitable. In this paper, we use a hypothetical phase III design to illustrate several statistical considerations in the designing stage of a confirmatory phase III trial. This discussion will include but not limited to selection of non-inferiority margin, determination of primary and secondary endpoints, dose selection, and multiplicity adjustment. We hope to use this case study to show the flow of statistical thinking in trial design and how to coordinate different requirements from different functions of a trial team.

More importantly, we also want to use this example to show how to use currently available tools to design flexible MTP that can fit one’s need and the caveat in choosing an MTP.

## A Hypothetical Phase III Clinical Trial Design

After the encouraging Phase II results are obtained from a drug candidate, the project team decided to progress it for Phase III development. The underlying disease is a difficult condition, and there was only one approved drug on the market. For Phase III study design, it is not ethical to conduct a placebo-controlled trial. Hence the Phase III program would have to be a non-inferiority trial designed to compare with the active control. For this indication, the primary endpoint is well established and widely accepted by regulatory agencies. In order to use this primary endpoint to design a non-inferiority trial, it is critically important to calculate the non-inferiority margin for this comparison. Such a margin will have to be agreed between the sponsor and the regulatory agencies, before the trial design can be finalized.

Based on the Phase II findings, a different endpoint demonstrates promising results. The project team considers this endpoint could distinguish the study drug from the active control, and other potential competitors which may be introduced to the market in some future time. On this basis, this endpoint needs to be included in the Phase III design as a key secondary endpoint. The hope is that if the primary endpoint demonstrates non-inferiority to the active control, meanwhile, this secondary endpoint could show superiority, then the study drug can be marketed with a strong label. A drug label contains important efficacy and safety information to assist physicians in prescribing the drug. Information contained in the drug label (3) informs patients regarding appropriate drug use and potential adverse effects. The drug label is finalized after a new drug completes the pre-marketing development process but at the time it receives regulatory approval and is ready for general patient population use. The sponsor and the regulatory agency (e.g., FDA) agree upon the content of the label. If the sponsor hopes to promote the study drug using a secondary endpoint, it is important for this secondary endpoint to be α-protected so that it could be included in the drug label if statistical significance can be observed.

For this development program, a target dose was selected based on all of the early findings including non-clinical and clinical Phase I and II data. This target dose is used in the Phase III study design to be compared with the active control. However, the project team also considered the risk that there might be late stage adverse events that may cause safety concerns at the target dose. Hence a lower dose was introduced and this study becomes a three-arm design – target dose of the study drug, lower dose of the study drug, and the active control.

Both the primary and the secondary endpoints are continuous variables calculated as change from baseline to the designed study time point. The distributions of residuals from both variables appear to follow normal distribution. Given the large sample size needed for the Phase III study, a linear model is proposed to analyze these endpoints. For both endpoints, a reduction from baseline indicates clinical improvement. Hence the clinical efficacy can be achieved in a placebo-controlled study if the mean treatment difference is negative, clinically meaningful, and statistically significant. For an active-controlled trial, efficacy can be achieved if the upper limit of the 95% confidence interval for mean difference (test drug subtract active control) does not exceed the pre-specified non-inferiority margin.

Given this three treatment group design, the project team hopes that the target dose will deliver clinical superiority to the active control (in the primary endpoint), and the lower dose will deliver at least non-inferiority in efficacy. If the safety profile for the target dose is acceptable, then the sponsor will market the study drug at the target dose. However, in case there is safety concern from the target dose, then if the low dose is relatively safe, and with non-inferiority in efficacy, this lower dose can be delivered to the general patient population.

For the secondary endpoint, a minimally clinically important difference (MCID) can be established using treatment difference of change from baseline to the specific time point. The difference is based on mean outcome of test drug subtract mean outcome of active control, and a negative value means treatment improvement. The project team expected that clinical superiority can be observed in the comparison of target dose against active control and also in the comparison of lower dose against the active control. Sample size calculation using the secondary endpoint is based on the known standard deviation, and this MCID.

## Statistical Considerations

Given this background, there are several statistical considerations for this Phase III study design – issues relating to non-inferiority, dose selection, and multiple endpoints. These issues are presented in more details below.

In order to establish the non-inferiority margin, previous comparative studies including both placebo and the active comparator were used. It would be helpful to review the existing data from these historical clinical trials, and obtain an estimate of treatment difference (of active comparator against placebo) from the combined data across these studies. The project team first identified those studies from literature, then a meta-analysis was performed on these studies. In each of the historical study, the efficacy measurements from the primary endpoint are obtained both from the placebo group, and from the active comparator. A meta-analysis with random-effect model was performed on these measurements and a 95% confidence interval was constructed on the mean treatment difference between the active comparator and the placebo.

**Figure 1:**
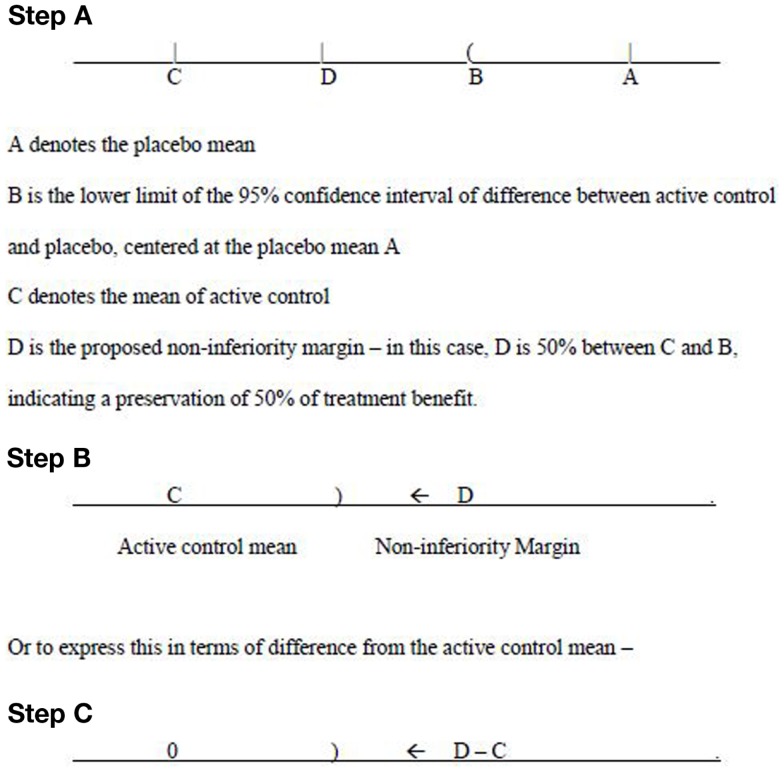
**Non-inferiority margin**.

The primary endpoint is a continuous variable, and negative value of change from baseline indicates improvement in efficacy. On this basis, the treatment difference of comparator mean subtracting placebo mean is a negative number. According to the 95–95 rule, in order to preserve 50% of treatment effect, the middle point (50% to the left of the lower confidence limit from the placebo mean) between the active control mean and the lower confidence limit is chosen as the non-inferiority margin – see Figure 1 (Step A).

The thinking process goes from the placebo mean (A), to the point B, where B is obtained from the calculation of 95% confidence interval on differences between active control and placebo. Then apply this confidence interval on the point A. Next to look at C, which is the mean of active control. Finally a non-inferiority margin D is selected. Suppose the interest is to preserve 67% (2/3) of treatment benefit, then D should be chosen to be 67% lower from B, or 33% above C. This means that for the Phase III study the mean difference of study drug effect subtract the active comparator effect is expected to have a negative value, and the upper limit of the corresponding 95% confidence interval should not exceed this non-inferiority margin.

The determination of such a margin should be based on extensive discussions with regulatory agencies. Only after the agreement is reached between the sponsor and the regulatory agencies, such a margin can then be used for the Phase III study design. Sample size calculation will be based on this margin. From Figure 1 (Steps B and C), the upper limit of 95% confidence interval – denoted as “)“ for the treatment difference (mean of study drug subtract mean of active control) needs to be below (to the left of) the margin (D −C) in order to claim non-inferiority. Of course, for superiority, the upper confidence limit should be below 0.

In a typical clinical development program, dose selection takes place during the Phase II development stage. However, in many clinical development programs, even with an extended Phase II exploration, often times only a range of active doses can be considered for Phase III confirmation. It is very rare that only one specific fixed dose is confidently proposed to bring into the Phase III development. Usually only a single target dose is suggested for the Phase III program. However, in many Phase III designs, a dose that is higher, or lower than the target dose (in some cases one higher dose and one lower dose are both included) is also tested. Sometimes both a higher dose, and a lower dose, in addition to the target dose are used in the Phase III development.

From drug label point of view, each approved dose for the study drug needs to be efficacious, and safe. Hence a given dose can only be approved if the benefit outweighs the risk. The project team hopes that the target dose will deliver an acceptable safety profile, and that both the primary and the secondary endpoint can demonstrate superiority to the active control. However, if the long-term safety events observed from the target dose is not as good as expected, then the lower dose is expected to serve as a backup. The lower dose is considered to be relatively safe, with superiority on the secondary endpoint, but may possibly only deliver non-inferiority to active control on the primary endpoint. Based on power calculation, the sample size to be used for demonstration of non-inferiority of the primary endpoint is sufficiently large to also provide good power for the secondary endpoint to show superiority. Therefore, sample size is calculated using the non-inferiority margin pre-specified for the primary endpoint.

From above discussion, it is clear that in order to control the Type I error (α), under non-inferiority, dose selection, and multiple endpoint issues, the major statistical concern in this study design is the MTP. The section below focuses on such a discussion.

## Multiple Testing Procedures

In designing a MTP for the program, it is all about how to assign and “recycle” the significance level. The idea of recycle has been applied in several MTP in the literature, such as the Holm procedure and Fallback procedure, etc. In the case of two null hypotheses, a procedure allowing recycle of the significance level will test one null hypothesis at α/2 and the other hypothesis at α if the first one has been rejected. This is improvement over the Bonferroni procedure which will test both hypotheses at α/2. This idea is viewed as recycling the significance level of α/2 after rejecting the first hypothesis.

In this specific drug development program, non-inferiority of the primary endpoint is in the first hierarchy of a sequence of hypotheses. Upon proving non-inferiority of the primary endpoint, the project team can either test the superiority of the secondary endpoint or pursue superiority for the primary endpoint. However, as discussed above, the two directions are both logically sound. Hung and Wang (1) argued that in this case the superiority of primary endpoint and secondary endpoint should be tested simultaneously and a MTP should be carefully designed to control the family-wise error rate.

First we design a tree-structured gatekeeping procedure for this case. There are in total six null hypotheses of interest as listed below:

- H_1_: high dose is inferior to active control in primary endpoint
- H_2_: low dose is inferior to active control in primary endpoint
- H_3_: high dose is not superior to active control in primary endpoint
- H_4_: high dose is not superior to active control in secondary endpoint
- H_5_: low dose is not superior to active control in primary endpoint
- H_6_: low dose is not superior to active control in secondary endpoint

Two families should be considered in the current setting, which are F1 = {H_1_, H_2_} and F2 = {H_3_, H_4_, H_5_, H_6_}. The first family F1 should be tested first. The second family F2 will be tested only if at least one rejection is observed in the first family. More specifically, we test H_3_ and H_4_ if and only if H_1_ is rejected, while H_5_ and H_6_ are tested if and only if H_2_ is rejected.

Gatekeeping procedure will test H_1_ and H_2_ as if no further families of hypotheses are of interest. Using Bonferroni adjustment, this requirement means the full α will be split between H_1_ and H_2_, usually α/2 for each when we weigh H_1_ and H_2_ equally. Upon showing significant result for H_1_, the significant level α/2 will then be passed to H_3_ and H_4_. Assuming equal weighting again, this α/2 will be split to α/4 for each of H_3_ and H_4_. The same procedure will be applied to H_2_, H_5_, and H_6_.

If one wishes to use a more powerful method such as Hochberg, it can be done for the second family among H_3_ and H_4_ (or H_5_ and H_6_). Instead of equally splitting α/2 between H_3_ and H_4_, one can compare the larger *p*-value of H_3_ and H_4_ with α/2 and reject both of the hypotheses if *p*-value <α/2. Otherwise compare the smaller *p*-value with α/4 and reject only the corresponding hypothesis with smaller *p*-value. However, the Hochberg procedure cannot be applied to the first family directly as it is “non-separable” according to Dmitrienko and Tamhane (4) and a truncated version of Hochberg procedure is recommended. On the other hand, the multistage and mixture parallel gatekeeping procedures proposed in the same paper does not work for this specific example because {H_1_, H_2_} is not a parallel set for the secondary family of hypotheses.

It is essential to know how much significant level can be passed from H_1_ to H_3_ and H_4_, and how much can be passed from H_2_ to H_5_ and H_6_. These two sets of significant levels are separate and cannot be shared between the two paths with regular tree-structured gatekeeping procedure.

**Figure 2:**
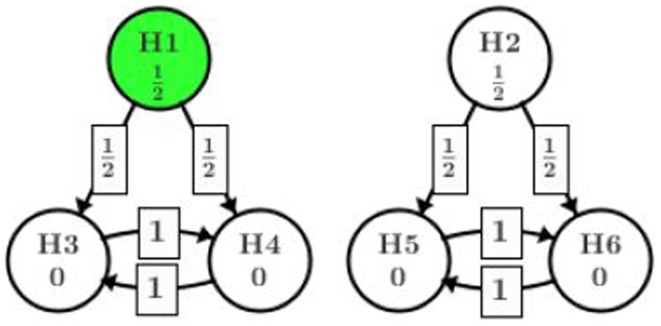
**Gatekeeping procedure**.

This tree-structured gatekeeping procedure assuming equal weighting can be visualized in Figure 2 using the new graphical representation proposed by Bretz et al. (5). R package “gMCP” is developed to implement the graphical approach which provides a graphical user interface. In this manuscript, we refer to the original paper by Bretz et al. (5) and use their R package to create the visualization of all examples. Interested readers can also reproduce the graphical approach steps for each example introduced in this manuscript and follow the dynamic illustration provided by the “gMCP” package.

Figure 2 indicates that at the very first step, the significant level α is split equally among H_1_ and H_2_. After this step, each part is bound with the significant level assigned. In case one path fails to show significance (for example, H_2_ is retained), the part of significant level assigned to that path is consumed. The arrows in the graph show how significant level will be shifted from one node to another given the hypothesis in the starting node is rejected at the assigned level. The weight on the arrow tells how much of the significant level in the starting node will go to the next node. In this example, H_1_ is originally assigned with significant level α/2. If H_1_ is rejected at α/2, then one half of the α/2 will be given to H_3_ and the other half to H_4_. Since H_3_ and H_4_ originally did not get any significant level assigned, they will both be tested at α/4 which comes from splitting the α/2 from H_1_. In the next step, if H_3_ is rejected at level α/4, then all of the value α/4 will be passed to H_4_ (and vise versa). Whether H_3_ or H_4_ should be tested first depends on their *p*-values. For more details on the graphical representation, we refer to the original paper by Bretz et al. (5). From this point on, we assume the readers have some familiarity to the graphical approach and can understand the graphical terminologies.

What worth to point out is that the tree-structured gatekeeping have two separate and independent paths, i.e., {H_1_, H_3_, and H_4_} and {H_2_, H_5_, and H_6_}. No significance level can be “recycled” and reassigned between the two paths. Intuitively, we should be able to test H_2_ at full α given H_1_ is rejected at α/2 as Holm’s procedure suggests. However, by adding the secondary family of hypotheses, this path is closed and H_2_ has to be tested at α/2 even though all of H_1_, H_3_, and H_4_ are rejected.

This observation roots in the principle that is used when constructing gatekeeping procedures, which is the testing result of secondary endpoints cannot affect the test on primary endpoints. The gatekeeping procedure (6) implemented this principle by not allowing reuse of the significant level from one path on the other paths. Their rationale is such reallocation will cause the low dose primary endpoint hypothesis H_2_ tested with an alpha level that depends on the testing result of high dose secondary family hypotheses (H_4_ and/or H_3_). On the other hand, there has been several publications suggest a different opinion that the principle should be restricted only within fixed dose level. Specifically, the testing result of H_2_ should not be affected by the test on H_5_ and H_6_ (all in the low dose path), but might benefit from rejection of both H_3_ and H_4_ (from high dose). Interested readers can refer to Wang and Cui (2) for a summary.

**Figure 3:**
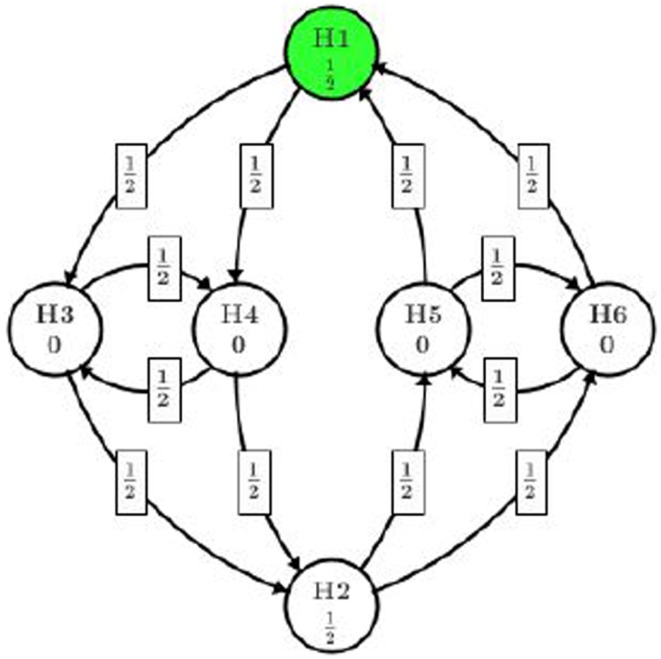
**Reuse alpha among dose levels**.

This new implementation allows us to test H_2_ at full α level if high dose shows efficacious on both endpoints (rejection of H_1_, H_3_, and H_4_), which is more powerful than the original gatekeeping procedure. The graphical representation of this implementation is illustrated in Figure 3.

In this procedure, all of the significant level α/2 assigned to the high dose path can be reused in low dose path if all H_1_, H_3_, and H_4_ are rejected. Even if only one of H_3_ or H_4_ is rejected along with H_1_, one eighth of α can be reused in low dose path.

Note that this approach also allows the project team to assign different initial significant levels to H_1_ and H_2_. For example, if the high dose is of more interest, the project team can assign three quarters of α to H_1_ instead of α/2 and only one quarter of α to H_2_. Since the trial will be powered to show significance at 3α/4 for H_1_, it is very likely that H_2_ will be tested at α with the recycled significant level from H_1_. Also in this way, the team is on the safe side to have a positive trial than testing H_1_ at α/2 level.

Users need to be careful writing their programs since this graphical approach is not yet supported in regular tree-structured gatekeeping procedure SAS macro. The graphical approach R package “gMCP” is recommended.

## Example

To better understand how the MTP worked, we consider a specific artificial scenario of the active-controlled trial with two doses and two endpoints example with several different approaches and discuss their difference in findings. The significant level is fixed at two-sided level 0.05.

Suppose the following raw *p*-values are observed for each null hypothesis:

- *p*_1_ = 0.005 for H_1_: high dose is inferior to active control in primary endpoint
- *p*_2_ = 0.027 for H_2_: low dose is inferior to active control in primary endpoint
- *p*_3_ = 0.020 for H_3_: high dose is not superior to active control in primary endpoint
- *p*_4_ = 0.009 for H_4_: high dose is not superior to active control in secondary endpoint
- *p*_5_ = 0.133 for H_5_: low dose is not superior to active control in primary endpoint
- *p*_6_ = 0.018 for H_6_: low dose is not superior to active control in secondary endpoint

First we apply the gatekeeping procedure. Since α will be split among H_1_ and H_2_, each will be tested at level α/2 = 0.025. In the first step, H_1_ is rejected and the significant level α/2 can be used again among H_3_ and H_4_. Since H_2_ is not rejected, no further test will be performed on H_5_ and H_6_ and the significant level of α/2 on H_2_ is consumed. In the second step, we test H_3_ and H_4_. If using the Bonferroni–Holm method, we follow the order that *p*_4_ < α/4 = 0.0125 and *p*_3_ < α/2 = 0.025, thus both H_3_ and H_4_ are rejected. If the Hochberg method is used, we reject both of them directed by comparing *p*_3_ < α/2 = 0.025.

As we mentioned before, even the high dose group shows significance in all hypotheses, the significant level in this family (H_1_, H_3_, and H_4_) cannot be reused by the low dose group. Thus, none of the low dose hypotheses are rejected by gatekeeping procedure. The testing result is summarized in Table 1.

**Table 1:** **Testing result of gatekeeping procedure and the two modified graphical approaches**.

	Gatekeeping	Figure 3	Figure 4
H_1_	Reject	Reject	Reject
H_2_		Reject	Reject
H_3_	Reject		Reject
H_4_	Reject	Reject	Reject
H_5_			
H_6_			Reject

To overcome the limitation of gatekeeping procedure, several authors have recommended to reuse significant level across different dose levels. Thanks to the flexibility of graphical approach, this idea can be specified as Figure 3. Now we explore how we can benefit from recycling the significant level. At the first step, H_1_ and H_2_ are of consideration and we start from the one with smaller *p*-value, which will be H_1_ in our case (marked in dark color). Since *p*_1_ < α/2 = 0.025, H_1_ will be rejected and its assigned significant level will be split among H_3_ and H_4_, each getting α/4 = 0.0125. Then we test H_4_ because *p*_4_ is smaller and reject it as *p*_4_ < α/4. The significant level used on H_4_ will then be split in half to H_3_ and H_2_, each getting α/8. This splitting results in overall 3α/8 on H_3_ and 5α/8 on H_2_. At this stage, whether H_3_ or H_2_ should be tested first depends on the ratio of *p*_3_/(3α/8) and *p*_2_/(5α/8), whichever is smaller. According to the observed *p*-values, *p*_2_/(5α/8) is smaller and H_2_ will be tested and rejected first. Then we move our attention from H_2_ to H_5_ and H_6_ and come back to H_3_ later. The significant level 5α/8 on H_2_ will be split to H_5_ and H_6_, each getting 5α/16. H_6_ with a smaller *p*-value will be tested first. However, since *p*_6_ > 5α/16 = 0.016, we fail to reject H_6_ or H_5_ and consumed the significant level on them. At the final step, we test H_3_ with the significant level 3α/8 assigned to it. Since *p*_3_ > 3α/8 = 0.019, we fail to reject H_3_ and conclude the whole process.

This procedure also ends up rejecting three out of six hypotheses, which is summarized in Table 1. One might argue that it is no better than the original gatekeeping procedure as we cannot quantify which hypothesis is of more importance and suggest the rejection of H_2_ is more important than the rejection of H_3_. One may also argue that, there is too much weight shifted to H_2_ after H_4_ is rejected. In practice, it makes more sense to complete one path H_1_ → (H_3_ and H_4_) of the high dose before splitting too much alpha to the other path. In fact, it is quite arbitrary to pass one half of the significance level from H_3_ to H_2_ and H_4_ (or H_4_ to H_2_ and H_3_).

**Figure 4:**
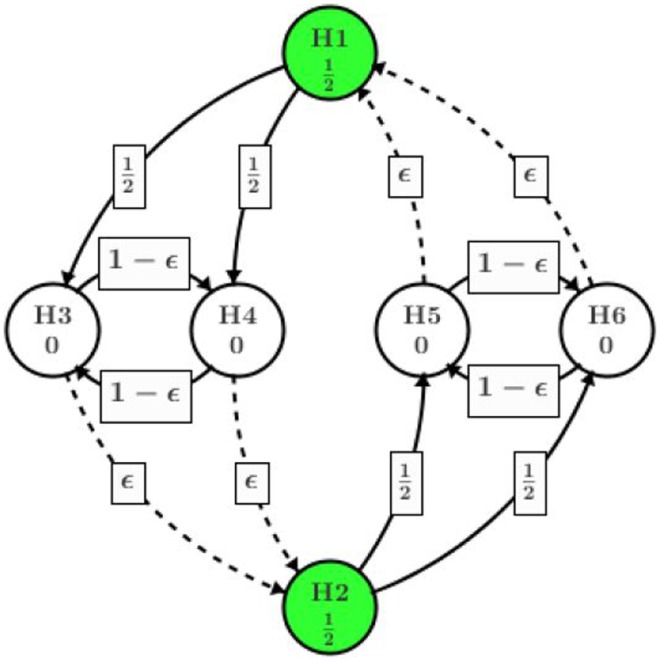
**Infinitesimal small weight edge at H_3_ (H_4_) TO H_2_**.

Alternatively, we present a better design in Figure 4 using the concept of infinitesimal small weight. Assume the trial team believes it is of more interest to show positive result in all high dose hypotheses (among H_1_, H_3_, and H_4_) before moving on to the low dose. An infinitesimal small weight epsilon edge can be assigned from H_3_ to H_2_ and H_4_ to H_2_.

Different from the procedure in Figure 3, after H_4_ is rejected at the second step, almost all of its significance level (except an infinitesimal amount) will be assigned to H_3_. Then H_3_ will be tested at level almost equal to α/2, which will be rejected as *p*_3_ = 0.020 < α/2. Then the significance level of α/2 will be passed to H_2_, adding to its original α/2 assigned at the beginning of the process. Next step H_2_ will be tested at level alpha and rejected since *p*_2_ = 0.027. H_5_ and H_6_ will receive α/2 each upon the rejection of H_2_. Eventually, H_6_ will be rejected and H_5_ retained. Overall, the procedure utilizing infinitesimal edge will have five null hypothesis rejected as summarized in Table 1.

**Figure 5:**
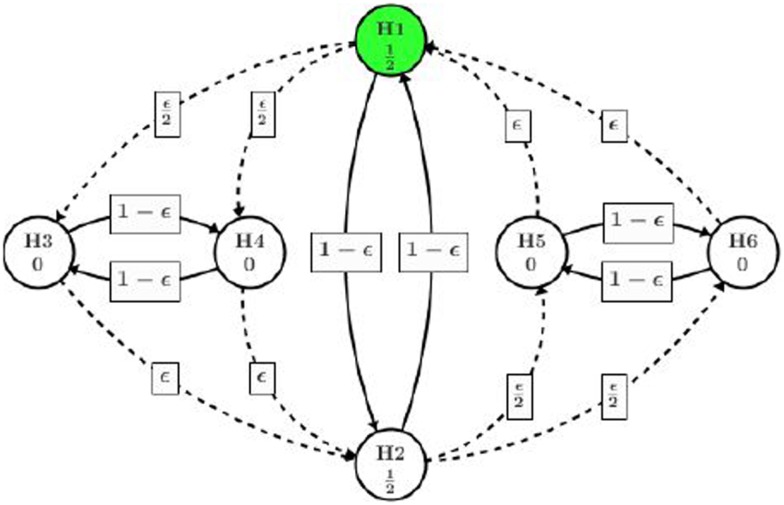
**Direct path between H_1_ and H_2_**.

Similarly, if it is more important to show both high dose and low dose are non-inferior at the primary endpoint (H_1_ and H_2_), one can add direct edge between H_1_ and H_2_ while replacing the rest of the edges with infinitesimal weight as shown in Figure 5. The graphical approach is flexible enough to adjust according to different requirement in clinical research.

It is also worth to point out that when there is a tie in the *p*-values, the graphical approach will always reach the same conclusion regardless which hypothesis involved in the tie will be tested and rejected first. In theory the probability to observe exactly equal *p*-values from two independent tests is 0, we do quite often in practice see *p*-values <0.0001 being reported in statistical software. The proof that graphical approach is robust with tied *p*-values can be found in Bretz et al. (7).

## Discussion

In clinical development of new medicinal products, alpha protection is one of the most important statistical tasks. The drug approvals and the go/no-go decisions are all based on the fact that the experiment-wise Type I error (alpha) not being inflated. When there are multiple treatment groups, multiple endpoints, or multiple objectives in a clinical trial, the pre-specification of MTP becomes a very important statistical practice in study design.

The process of designing a Phase III clinical trial involves in many discussions among team members – statisticians, clinicians, regulatory affairs, marketing, medical affairs – and their corresponding line management as well as the upper management within the sponsor. In many situations, the protocol may even be sent to FDA and/or other regulatory agencies to seek for their input. At this point, choice of the MTP strategy is critical in addressing the primary and secondary objectives under the given study design assumptions. Different assumptions made during this stage will have different implications after the data read out. Therefore, various MTP strategies need to be communicated clearly from the statisticians to other team members and the upper management, benefits and risks of each strategy need to be fully discussed before a final set of multiple comparison adjustments can be selected.

This manuscript covers statistical considerations in designing a Phase III active-controlled trial with two doses and two endpoints. Here we present the selection of MTP under three challenges: non-inferiority/superiority testing, multiple doses and multiple endpoints. An example is introduced here. This hypothetical, practical example covers the gatekeeping and the graphic approaches to deal with these challenges. As can be found from this example, various assumptions or various priorities of objectives can lead to various MTP strategies. From a statistical application point of view, we hope to make the proposal to be flexible so that the statistical method can be applied to a wide range of situations in solving real world problems.

These approaches, as well as the thinking behind them, can be generalized to cases where there are more than two doses or more than two endpoints or both. Furthermore, when a case that is simpler than this setting is proposed as when only one or two of the three (non-inferiority, multiple doses, multiple endpoints) challenges appear, the thinking behind our proposal can still be applicable. Although the situation is derived from a continuous variable which serves as the primary and secondary endpoints, the discussion covered in this manuscript can also be applied to binary data, count data, or time-to-event data.

## Conflict of Interest Statement

The authors declare that the research was conducted in the absence of any commercial or financial relationships that could be construed as a potential conflict of interest.

## References

[1] Hung J Wang S Some controversial multiple testing problems in regulatory applications. J Biopharm Stat (2009) 19:1–11 10.1080/10543400802541693 19127460

[2] Wang B Cui X A new partition testing strategy for multiple endpoints. Stat Med (2012) 31(20):2151–68 10.1002/sim.5366 22532094

[3] Ting N Dose Finding in Drug Development (Chapter 1). New York: Springer (2006).

[4] Dmitrienko A Tamhane A Mixtures of multiple testing procedures for gatekeeping applications in clinical trials. Stat Med (2011) 30:1473–88 10.1002/sim.4008 21503948

[5] Bretz F Maurer W Brannath W Posch M A graphical approach to sequentially rejective multiple test procedures. Stat Med (2009) 28:586–604 10.1002/sim.3495 19051220

[6] Dmitrienko A Offen W Westfall P Gatekeeping strategies for clinical trials that do not require all primary effects to be significant. Stat Med (2003) 22:2387–400 10.1002/sim.1526 12872297

[7] Bretz F Posch M Glimm E Klinglmueller F Maurer W Rohmeyer K Graphical approaches for multiple comparison procedures using weighted Bonferroni, Simes, or parametric tests. Biom J (2011) 53(6):894–913 10.1002/bimj.201000239 21837623 PMC3427907

